# Dealing with great challenges via rigorous and relevant empirical sport management research

**DOI:** 10.3389/fspor.2023.1180710

**Published:** 2023-04-03

**Authors:** Joerg Koenigstorfer

**Affiliations:** Department of Sport and Health Sciences, Technical University of Munich, Munich, Germany

**Keywords:** validity, theory, methodology, grand societal challenges, sports management

## Introduction

What are the great challenges in empirical sport management research to come? Arguably, each sport management researcher (group) will face their own challenges when designing and executing studies and analyzing the data. Having said this, I do believe that there are common challenges that affect the whole field of sport management research. In this article, I want to summarize some of the key challenges as well as provide guidance and examples for how sport management researchers can cope with these challenges in empirical studies. This is important, because empirical studies are often the core of publications in scientific journal and the dissemination to the wider public.

## Great challenges in sport management research

In empirical research, we aim for *rigor*, that is, high validity, and *relevance*, that is, high importance to decision-makers. In what follows, I focus on great challenges that I feel are relevant to secure both the rigor and the relevance of empirical studies. In what follows, I want to describe three challenges: Challenge I, focusing on theory; Challenge II, focusing on methodology; and Challenge III, focusing on substantive contributions of empirical studies.

### Challenge I: The need for theoretical advancement

In sport management research today, the use of existing theories that were developed in allied disciplines seems to be predominant, most likely due to the perceived lack of need to make adaptions, extend the theory, or generate new theories, but also due to the relatively few theories that have their origins in sport management and that are indeed peculiar to the sport management discipline. But what are the limitations of the theories that have been developed in allied disciplines, such as social psychology, human resources, and organizational behavior? Sometimes, they do not provide sufficient answers to those who are closest to the problem (here: sport managers or athletes). If this was true, we should employ more deductive approaches (theoretical modeling) or inductive approaches [Grounded Theory, in particular ethnography, case study, and theory in use; see (1), for an example from marketing] to propose particular sport management theories, such as presented by Green (2) or Funk (3). Because of the applied nature of the field, sport management theories may reduce theory-practice gaps, particularly when the researchers who are engaged in the process of theory development consider theory and practice as inseparable (4). Sport management theories can be recommended to focus on the unique characteristics of sport (5, 6) and solve any discontent with existing conceptualizations towards high consistency with managerial reality (7). Research- and practice-oriented theories also provide value to educate students (8).

In academia, the central contribution to theory is visible in empirical studies that question existing theories, particularly in regard to how variables are related to each other (with a focus on practices or mechanisms) and what the contingencies or boundary conditions are. While some researchers may prefer the rather eclectic use of theories, which has the advantage of being able to look at topics from different conceptual angles, the eclectic use of theories often reduces the likelihood that particular theories are developed further. Yet, the critical discourse of particular theories with enough food for discussion and focus on detail is needed to advance the field: “good theory is practical precisely because it advances knowledge in a scientific discipline, guides research toward crucial questions, and enlightens the profession of management” (9). Thus, there is a need to carefully work with, or develop, theories from inside or outside sport management.

Inside the field of sport management, the three leading journals, *Journal of Sport Management*, *European Sport Management Quarterly*, and *Sport Management Review*, as well as up-and-coming journals, such as the *Journal of Global Sport Management* and *Frontiers in Sports and Active Living*, provide researchers with platforms to publish theoretically relevant empirical studies. Yet, most publications in these journals do not get cited by authors who publish in leading journals of the basic-science fields, such as management and economics, sociology, and psychology (e.g., the Financial Times 50 management journals that are particularly relevant to business schools). Good examples for excellent use and extension of theory, and prominent placement of their work within the basic-science fields, are the publication by Cornwell et al. (10), who advance Memory Theory by considering the associative strength between sponsorship cues, depending on articulation and whether a competitor is salient or not, Constandt et al. (11), who advance the Theory of Moral Development by showing that the presence of ethical codes within soccer clubs *per se* does not explain the evolution of ethical climate within the clubs, but that professionalization, sponsor presence, the support of whistleblowers, and helpdesks have a positive influence, as well as Berendt et al. (12), who show that the potential demise of an outgroup can make ingroups want to preserve the outgroup, thereby providing new insights into Social Comparison. Such articles can be expected to have a strong impact in various fields.

### Challenge II: The need for methodological rigor

Rarely, sport management research is cited for its methodological advancement, despite the potential of the field to provide worthwhile settings to innovate methodological procedures. Developing methodology further requires that we ask ourselves how we can get truly valid findings. One avenue is to move beyond simple correlational analyses, as indicated by Borsboom et al. (13): “correlations are epistemologically relevant because they are sometimes indicative of causality, but they are not, and cannot be, constitutive of validity”. Another avenue is the reliance on technological innovations from other fields to develop methodologies further, such as seen in positioning systems, face recognition, and consumer wearables that allow to track physiological parameters, and use extensions or combinations of these technologies to answer research questions. Field experiments, such as the one conducted by Mousa (14), are only one example of how to strengthen the level of evidence (particularly in regard to external validity). More good example for making a specific methodological contribution can be seen in DeSarbo et al.'s (15) work on parametric constrained segmentation of sport fans, or Stadler Blank et al.'s (16) work on the development of a team personality scale (i.e., a scale that performs better than previous scales and takes into account common method variance).

Methodological rigor is indicated by the correct use of state-of-the-art methodology. This is sometimes not the case, as argued by Cunningham and Ahn (17), who took a close look at the statistical procedures used in empirical sport management studies. They reveal that 16% of the moderation analyses in the top-three sport management journals are incorrect. Also, they state that “sport management researchers are relatively unlikely to specify moderators in their theoretical models or to test moderation in their analyses” and argue for more robust testing of moderation to inform theory. A good example of a rigorous testing of moderators can be seen in Berendt et al.'s (12) work. One commonality in this paper and in the papers cited above is that they present all the methods-related information that is needed to understand the findings. Such information is not only needed for replication analyses or studies, but also for meta-analyses, for example.

In empirical studies, researchers often wonder whether they should use one particular methodology (typically one that they are trained in and comfortable with) or a mix of methodologies. The mix of methodologies can be seen in the sampling strategy (e.g., recruiting members of online panels vs. athletes on-site), research design (e.g., conducting surveys vs. observations), and data analysis (e.g., running models with lower or higher flexibility), to name but some examples. To increase the rigor of the empirical work, a methodological mix (i.e., high pluralism) is desirable (18). For example, if researchers show that they get similar results in the laboratory and in the field, and for different samples, the validity of the results should increase. To do this, sport management researchers will be more and more challenged to conduct multiple studies (instead of one only), and be trained in multiple methodologies (or work in teams with different methodological expertise). Also, longitudinal studies may have particular value [as seen in (11)], particularly in regard to studying true cause-and-effect relationships. When doing empirical studies, researchers may adhere to the FAIR principles—Findability, Accessibility, Interoperability, and Reusability (19)—in order to promote the reuse of data. Also, researchers will be more and more asked to commit to open-science principles and practices, such as preregistering empirical studies, submitting data and codes as well as study materials along with manuscripts, and doing extensive robustness checks and ruling out alternative explanations in their analyses (e.g., *via* providing the relevant information in an Online Appendix). When following such an approach, not only training in state-of-the-art practices is needed, but also time (because more analyses are needed).

### Challenge III: The need for substantive contributions towards mastering grand societal challenges

Ideally, real-world problems studied by sport management researchers can be categorized into a larger societal grand challenge, which can at least partly be addressed in the particular research study. Given the breadth of actors (and hence, problems) in the sport system, solutions to societal grand challenges have the following characteristics: they tackle an important problem; they help the society develop for the better; and they often have global impact. Thus, not only managers should be the main target group of the research studies and their implications, but also public policy makers, who partly speak for those that have no voice [as seen for flora and fauna—important parts of the environment that often have no advocate; see (20), for example]. Areas in sport management with great need for substantive advancement for the greater good are: anti-doping, anti-corruption, promotion of human rights, sustainable development, sport-for-all, and public health, to name but a few, yet important fields of research.

Publicly funded research grants will likely be given preferably to those that provide solutions to societal grand challenges as opposed to small-scale problems of selected individuals or institutions. This is because these research grants are indirectly financed by tax payers, who expect that the grant is used to improve the society at large, such as in relation to sustainable development and peace. Sport management researchers will be more and more asked what “they bring to the table” and what else they need to do to truly help master the societal grand challenges. While some might argue that physical activity and sport—and thus, managing sport—inherently contribute to Sustainable Development (SDG) Goal 3 (21), not all sport management-related practices help achieve SDG 3. Racial or sexual discrimination and corrupted events are only some examples of negative effects on health, sustainability, and important human rights [e.g. (22) who focus on corrupted events]. More research is needed on how to prevent and reduce harmful practices so that physical activity and sport truly increase SDG 3-related positive outcomes, and what the mechanisms and practices as well as boundary conditions and contingencies are.

## Conclusion

By providing insights into what I perceive some of the great challenges and offering some solutions, I hope to inspire researchers to overcome these challenges in their future work and contribute to knowledge. While everyone naturally has different opinions of what research topics are interesting and what topics are not, there is agreement that the rigor and the relevance of the studies should increase the evidence level of our work and should make the sport management field more trustworthy towards researchers from other fields and decision-makers. The focus on these aspects should also make the work more enjoyable and increase the perception of a researcher's self-efficacy.
