# Corrigendum: Dealing with great challenges via rigorous and relevant empirical sport management research

**DOI:** 10.3389/fspor.2023.1234933

**Published:** 2023-06-21

**Authors:** Joerg Koenigstorfer

**Affiliations:** Department of Sport and Health Sciences, Technical University of Munich, Munich, Germany

**Keywords:** validity, theory, methodology, grand societal challenges, sports management

A Corrigendum on Dealing with great challenges via rigorous and relevant empirical sport management research By Koenigstorfer J. (2023) Front. Sports Act. Living 5:1180710. doi: 10.3389/fspor.2023.1180710

**Author Name Misspelled in Reference**

In the published article, the last name of one of the cited authors was incomplete in reference 16. The last name was given as “Blank”. It should be “Stadler Blank”. The corrected reference is:

“16. Stadler Blank A, Koenigstorfer J, Baumgartner H. Sport team personality: it's not all about winning!. *Sport Manage Rev*. (2018) 21(2):114–32. doi: 10.1016/j.smr.2017.05.004”

The citation in Great challenges in sport management research, *Challenge II: The need for methodological rigor*, paragraph one, has been changed from “Blank et al.'s (16)” to “Stadler Blank et al.'s (16)”.

The author apologizes for this error and states that this does not change the scientific conclusions of the article in any way. The original article has been updated.

